# Herbert Astley Knight

**Published:** 1908-12

**Authors:** 


### HERBERT ASTLEY KNIGHT, M.B., B.Ch., F.R.C.S. Edin., D.P.H., I.M.S.

We regret to record the death of Herbert Astley Knight, M.B., B.Ch., F.R.C.S. Edin., D.P.H., of the Indian Medical Service. He was stricken by cholera while on duty in Mooltan, India, in the first week of September, and died in a few hours. He was only 28. After finishing at the Royal High School, Edinburgh, he entered Edinburgh University in 1896. In 1902 he graduated, and in 1903 took the Diploma of Public Health. For two years he was House Surgeon in Weston-super-Mare, and for one year was House Surgeon to Bristol General Hospital. In 1906 he received a Fellowship of the Royal College of Surgeons of Edinburgh, and in the same year entered the Indian Medical Service. He was first stationed at Poona, and later transferred to Ambala, where he was put in charge of the Brigade Laboratory for the

Prevention of Disease. The Indian Government had in contemplation to appoint Lieutenant Knight when he would have attained his captaincy, a few months hence, to do special sanitary work in the Bombay Presidency.—*Medical Press and Circular.*

The fellow-students and friends of Dr. Knight in Bristol will receive the above information with much regret.

---

### Local Medical Notes.

